# Inequalities in Rotavirus Vaccine Uptake in Ethiopia: A Decomposition Analysis

**DOI:** 10.3390/ijerph17082696

**Published:** 2020-04-14

**Authors:** Abrham Wondimu, Jurjen van der Schans, Marinus van Hulst, Maarten J. Postma

**Affiliations:** 1 Department of Pharmaceutics, School of Pharmacy, College of Medicine and Health Sciences, University of Gondar, P.O. Box 196 Gondar, Ethiopia; 2 Department of Health Sciences, University Medical Center Groningen (UMCG), University of Groningen, 9713 AV Groningen, The Netherlands; j.van.der.schans@rug.nl (J.v.d.S.); r.hulst@mzh.nl (M.v.H.); m.j.postma@rug.nl (M.J.P.); 3 Department of Economics, Econometrics & Finance, Faculty of Economics & Business, University of Groningen, 9747 AE Groningen, The Netherlands; 4 Department of Clinical Pharmacy and Toxicology, Martini Hospital, 9728 NT Groningen, The Netherlands; 5 Unit of Pharmaco Therapy, Epidemiology & Economics (PTE2), Department of Pharmacy, University of Groningen, 9713 AV Groningen, The Netherlands; 6 Department of Pharmacology and Therapy, Faculty of Medicine, Universitas Airlangga, Surabaya 60132, Indonesia

**Keywords:** inequalities, rotavirus vaccine, uptake, concentration curve, concentration index, decomposition analysis, Ethiopia

## Abstract

A previous study in Ethiopia reported significant variation in rotavirus vaccine uptake across socioeconomic strata. This study aims to quantify socioeconomic inequality of rotavirus vaccine uptake in Ethiopia and to identify the contributing factors for the inequality. The concentration curve (CC) and the Erreygers Normalized Concentration Index (ECI) were used to assess the socioeconomic related inequality in rotavirus vaccine uptake using data from the 2016 Ethiopian Demographic and Health Survey. Decomposition analysis was conducted to identify the drivers of inequalities. The CC for rotavirus vaccine uptake lay below the line of equality and the ECI was 0.270 (*p* < 0.001) indicating that uptake of rotavirus vaccine in Ethiopia was significantly concentrated among children from families with better socioeconomic status. The decomposition analysis showed that underlining inequalities in maternal health care services utilization, including antenatal care use (18.4%) and institutional delivery (8.1%), exposure to media (12.8%), and maternal educational level (9.7%) were responsible for the majority of observed inequalities in the uptake of rotavirus vaccine. The findings suggested that there is significant socioeconomic inequality in rotavirus vaccine uptake in Ethiopia. Multi-sectoral actions are required to reduce the inequalities, inclusive increasing maternal health care services, and educational attainments among economically disadvantaged mothers.

## 1. Introduction

Vaccination is one of the most effective and efficient preventive health interventions worldwide [1]. Currently, vaccination averts 2–3 million mortalities each year and is expected to play a role in attaining 14 of the 17 Sustainable Development Goals [2,3]. However, vaccine-preventable diseases still claim 1.5 million lives annually because of poor immunization coverage. Globally, the World Health Organization (WHO) estimated that around 20 million children missed basic vaccinations in 2017 and nearly 60% of these children reside in Afghanistan, Angola, the Democratic Republic of the Congo, Ethiopia, India, Indonesia, Iraq, Nigeria, Pakistan, and South Africa [4]. In particular, our present paper focusses on Ethiopia where vaccine-preventable diseases such as diarrheal disease, lower respiratory tract infection, measles, and meningitis are the main causes of under-five mortality [5].

In 2015, rotavirus infection was responsible for 29.3% of all diarrheal deaths among children younger than 5 years across the world [5]. Consequently, vaccination against rotavirus has been introduced in several countries to reduce the health and economic burden associated with the rotavirus infection. Indeed, it is estimated that at global coverage of 27.8%, the rotavirus vaccine has already averted 28,800 deaths among under-5 children in 2016 and an additional 83,200 mortalities could have been prevented if the vaccine coverage was 100% [6]. It has been documented that after the introduction of rotavirus vaccine into national immunization programs, a decline in diarrhea-related hospitalizations was observed among children in Ethiopia and several other Sub-Saharan African countries [7,8].

Immunization services are underutilized in most developing countries, and socio-economic related inequalities in the immunization uptake may present additional obstacles for universal coverage of vaccines. Determinants of childhood immunization coverage in developing countries are numerous and complex [9]. The characteristics of the family, including educational and socio-economic status, are important predictors. Factors associated with the immunization system, such as physical access to the health care system, availability of health care workers, and direct and indirect costs of vaccination, also have a significant impact on the uptake of vaccines [10]. Several studies in developing countries documented inequality in immunization coverage favoring the rich [11,12,13,14]. However, the poorest would likely benefit the most from adequate vaccine coverage as they are more vulnerable to vaccine-preventable diseases that can potentially lead to severe health consequences as well as catastrophic and impoverishing health expenditures [15,16]. Hence, there is a need to assess vaccination inequality and tackle potential causes of inequalities to achieve better and potentially more fairly distributed health outcomes.

According to the 2016 Ethiopian Demographic and Health Survey (EDHS), only 33% of children aged 12–23 months received all age appropriate vaccines, with the highest coverage for the first dose of polio vaccine (81%). Among children aged 12–23 months in Ethiopia, 64.0% and 56.0% received the first and second dose of rotavirus vaccine (Rotarix^®^), respectively (with 2 doses reflecting the full schedule) [17]. The previous study in Ethiopia found that the uptake of rotavirus vaccine among children aged 12–23 months was significantly associated with the wealth index [18]. However, the study only investigated the direction of the association. To date, no studies have been conducted to quantify wealth-related inequality in the uptake of rotavirus vaccines in Ethiopia using the 2016 EDHS data.

The aim of this study is therefore to quantify the socioeconomic inequalities associated with the uptake of rotavirus vaccine in Ethiopia and to identify the factors contributing to inequalities. This will provide valuable information to design targeted strategies and policies to overcome potential disparities that exist in rotavirus vaccine coverage in Ethiopia and similar countries for that matter. Finally, analyses to identify drivers of the inequalities in rotavirus vaccine uptake can also provide transferable lessons for other vaccine programs within Ethiopia and potentially in other low/middle-income countries.

## 2. Methods

### 2.1. Data and Variables

The data used in this study was derived from the EDHS conducted in 2016. The survey was conducted with the main aim of providing a wide-ranging overview of maternal and child health issues in Ethiopia. In the EDHS 2016, a sample of 16,650 residential households was selected in two stages. Enumeration areas were selected with probabilities proportional to size followed by systematic sampling of households from each enumeration area with an equal probability. Interviews were completed with a total of 15,683 women aged 15–49 years old from the selected households. The women were interviewed on a range of socio-demographic and health issues, including childhood vaccination status [17].

In this study, the outcome variable was rotavirus vaccine uptake for children aged 12–23 months (1 if the child is vaccinated with two doses of rotavirus vaccine, 0 otherwise). Taking into account the reduced effectiveness of the rotavirus vaccine in developing countries setting, we considered that only children who took the full schedule (two doses in our case) were fully immunized. The following independent variables were selected based on previous studies in other African countries [11,14] and their availability in the dataset: maternal age (15–19, 20–34, 35–49 years old), maternal educational level (no education, primary, secondary, and higher), mother’s employment status (working, not working), maternal media exposure which is defined as listening to radio or reading newspaper or magazine, or watching television at least once a week (yes, no), religion (Orthodox Christian, Protestant, Muslim, others), residence place (urban, rural), region (Emerging regions, Established regions, Addis Ababa and Dire Dawa), antenatal care use (yes, no), institutional delivery (yes, no), baby postnatal check within 2 months after birth (yes, no), child sex (male, female), distance to health facility (not big problem, big problem), and partner’s educational level (no education, primary, secondary, and higher).

As mentioned above the administrative regions were grouped into three categories (Emerging regions, Established regions, Addis Ababa and Dire Dawa) based on their relative development profile and economic characteristics. Established regions consist of Tigray, Amhara, Oromia, Southern Nation Nationalities and People (SNNP), and Harari which are relatively developed regions whereas the Afar, Somali, Benishangul-Gumuz, and Gambela are categorized together as emerging regions being less developed and mainly characterized as pastoral communities. Addis Ababa and Dire Dawa city administrations are grouped together as they are urban centers with better infrastructures [19].

### 2.2. Measurement of Socioeconomic Status

The EDHS lack direct measures of living standard such as income and consumption data. Instead, the wealth index is used to measure the relative socioeconomic position of a given household. The wealth index is commonly computed using country data from the Demographic and Health Survey on assets ownership and housing characteristics such as a source of drinking water, type of toilet facilities, type of cooking fuel, materials used for housing construction, land ownership, and other assets [20]. In our present study, the wealth index provided in the EDHS was used as a proxy measure to socioeconomic status [17].

### 2.3. Measuring Inequality in Rotavirus Vaccination

**Figure 1:**
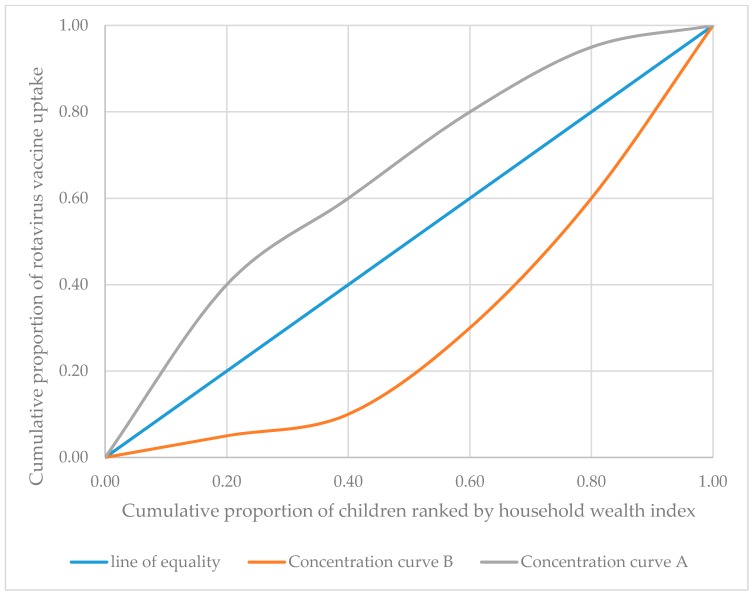
Hypothetical concentration curves for rotavirus vaccine uptake.

The degree of wealth-based inequality in rotavirus vaccination among children aged 12–23 months was measured by using the concentration curve (CC) and concentration index (CI) [21]. The CC plots the cumulative proportion of the rotavirus vaccination in the y-axis against the cumulative proportion of the population ranked by wealth index ranked from the poorest to the richest in the x-axis. If CC lies below the line of equality, a 45° straight line that passes through the origin, it suggests that the rotavirus vaccination is disproportionally concentrated among children that belong to the rich and vice versa. The interpretation of CC is illustrated using hypothetical CCs shown in Figure 1. The rotavirus vaccine uptake is proportionally distributed along the 45° line (line of equality) such that 40% of the rotavirus vaccine uptake is distributed among 40% of the population and so on. Whereas concentration curve ‘A’ shows pro-poor inequality. As shown by concentration curve ‘A’, for example, 60% of the rotavirus vaccination uptake is distributed among the poorest 40% of the population. On the other hand, concentration curve ‘B’ illustrates pro-rich inequality, with only 10% of the uptake is distributed among the poorest 40% meaning the remaining 90% of the vaccine uptake is distributed among the remaining 60% of the population.

Even though CC gives a graphical view of the inequality; it failed to provide the numerical quantity of the magnitude of the inequality. Therefore; the CI index was used for computing the degree of wealth-related inequality in rotavirus vaccine uptake among children aged 12–23 months in Ethiopia [21,22]. Taking into consideration that the outcome variable (rotavirus vaccine uptake) is binary, Erreygers’ normalized concentration index (ECI), which is suggested for such a condition, was used in our study to estimate the degree of inequality in the uptake of rotavirus vaccine [23,24,25,26,27,28,29,30,31].

The ECI can be expressed as:

(1) $$ECI=\frac{8*cov\;\left(y_{i},\;r_{i}\right)}{b-a}$$

where *y_i_* is rotavirus vaccine uptake, *r_i_* is the socioeconomic status ranking of individual *i* by wealth index, *cov* is covariance, and ‘*b*’ and ‘*a*’ represents the upper and lower bound of the outcome variable, respectively. The range (*b*–*a*) becomes one for binary variables, like in our case.

The value of the ECI can vary between −1 and +1; where the negative (positive) value shows rotavirus vaccine uptake is concentrated more among people with lower (higher) socioeconomic status, and ECI takes the value of zero in the absence of any socio-economic related inequality in rotavirus vaccine uptake. The absolute value of the ECI provides information about the magnitude of the observed inequality.

### 2.4. Decomposition Analysis

Although the ECI of rotavirus vaccine uptake shows the extent of socioeconomic inequality in the country, it does not provide evidence of possible factors contributing to the observed inequalities. Identifying these determining factors is instrumental to design relevant policy measures. In this study, therefore, decomposition analysis of ECI was employed to identify factors contributing to the inequality of rotavirus vaccine uptake [21,32].

Suppose the outcome variable of interest, rotavirus vaccine uptake (*y_i_*), is defined as a linear function of the explanatory variables according to the following multivariate linear regression equation:

(2) $$y_{i}=\alpha +\sum_{k}\beta_{k}x_{ki}+\varepsilon_{i}$$

where

*y_i_* is rotavirus vaccine uptake (*y_i_* = 1 if the child took two doses of rotavirus vaccine and *y_i_* = 0 if not);
*x_ki_*: a set of *k* explanatory variables for rotavirus vaccine uptake;
*β_k_*: regression coefficients of explanatory variables *x_k_*;
*ε_i_*: error term.

Then, the ECI for rotavirus vaccine uptake can be decomposed into the contribution of an individual explanatory variable by using Equation (3) [32].

(3) $$\mathrm{ECI}=4\left[\underset{k}{\sum }\beta_{k}{\bar{x}}_{k}C_{k}+GC_{\varepsilon }\right]$$

where *β_k_* is the coefficient of explanatory variables estimated from linear regression given in Equation (3), $\bar{X}$*_k_* is the mean of the explanatory variable (*x_k_*), *C_k_* is the concentration index of the explanatory variables, and *GC_ε_* is a generalized concentration index for the error term (*ε*).

As indicated in Equation (3), the ECI is cleaved into a deterministic and a residual component. The deterministic component, $\sum_{k}\beta_{k}{\bar{x}}_{k}C_{k\;}$, consists of a sum of the contribution of each explanatory variable to inequality in rotavirus vaccination status. The contribution of a given explanatory variable (*x_ki_*) to the inequality depends on the degree to which how it is distributed by socioeconomic status (measured by its concentration index, *C_k_*) and how it is associated with rotavirus vaccine uptake (reflected by its regression coefficient, *β_k_*). The higher *C_k_* or $\beta_{k}$ of the explanatory variable, the higher its contribution to the observed overall inequality. The percentage contribution of each explanatory variable to the overall inequality was obtained by dividing its contribution by the ECI and multiplying by one hundred. The residual component in Equation (3), $GC_{\varepsilon }$, reflects the inequality of rotavirus vaccination that cannot be explained by the explanatory variables included in the model. Even though binary variables are best estimated by non-linear models, a linear model was fitted to our data in Equation (3) as a linearity assumption of decomposition analysis is fulfilled and the results are easier to interpret with the latter one. For checking robustness, we repeated the decomposition analysis using partial effects of probit regression and found fairly consistent results, and the pattern remained unchanged. This is in line with what was previously reported [33,34]. Hence, the results of the linear model are presented in this study.

Erreygers and Kessels [35] argued against including socioeconomic status as an independent variable in the regression of health during decomposition analysis. When included, the residual component will be close to zero and the socioeconomic status itself explains the majority of the socioeconomic related inequality of health. However, this can be considered as an artifact that results from a high degree of correlation between the socioeconomic status variable used both as an independent variable in regression of health and a ranking variable for estimating the concentration index of health. Accordingly, in our study, it was decided not to use the socioeconomic variable as an explanatory variable in the regression model shown in Equation (3).

During analysis, sampling weights provided with the EDHS dataset were used. The variance inflation factor (VIF) was calculated to assess multicollinearity in the model. All VIF values were less than 10 indicating the absence of multicollinearity among variables [36]. Bootstrap approach with 1000 replications was used to obtain standard errors. The statistical analyses were performed in STATA version 16 (StataCorp, College Station, TX, USA).

## 3. Results

### 3.1. Descriptive Statistics

**Figure 2:**
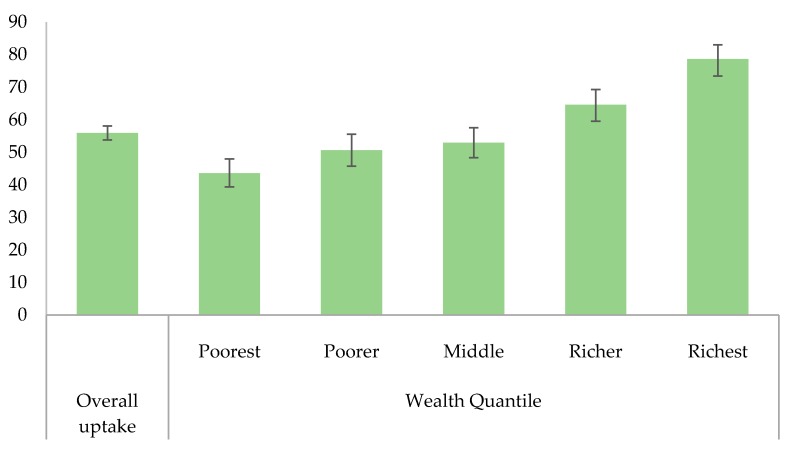
Distribution of rotavirus uptake among children aged 12–23 months in Ethiopia, 2016 EDHS (The error bar shows a 95% confidence interval).

Table 1 shows the characteristics of children aged 12–23 months who were included in this study. The majority of the children were from rural areas (88.4%) and the Established regions (90.9%). About 46% of the children were male and a quarter of the children belonged to the poorest household. Rotavirus vaccination coverage is shown in Figure 2. About 56% of the children received the full schedule (2 doses) of the rotavirus vaccine. The proportion of vaccinated children with a full schedule of rotavirus vaccine varied across different socioeconomic statuses. The highest rotavirus vaccine coverage (78.6%) was reported among children in the wealthiest households, while the lowest was reported among children in the poorest households (43.6%).

### 3.2. Wealth-Related Inequality of Rotavirus Vaccine Uptake

**Figure 3:**
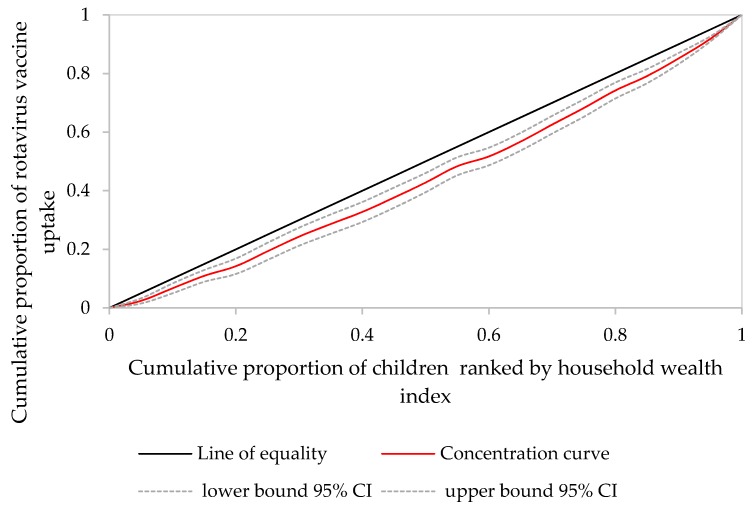
Concentration curve for rotavirus vaccine uptake among children aged 12–23 months in Ethiopia, 2016 EDHS.

Figure 3 depicts CC for rotavirus vaccination uptake among children aged 12–23 months in Ethiopia. The CC lay below the line of equality and, as presented in Table 2, ECI was found to be 0.270 (*p* < 0.001) indicating a pro-wealthy inequality of rotavirus vaccination in Ethiopia.

### 3.3. Results of Decomposition Analysis

The results of the decomposition analysis are depicted in Table 2. As shown in the coefficient column, Muslim religion was associated significantly with lower rotavirus vaccination uptake while antenatal care use was associated significantly with higher uptake of the vaccine. The CI column demonstrates how each independent variable is distributed across socio-economic status. The vast majority of the explanatory variables considered in this study, including protestant religion, antenatal care use, institutional delivery, and exposure to media were concentrated more among the relatively wealthier segment of the population as demonstrated by their significant positive CI. In contrast, variables such as mothers in the 20–34 years age group, Muslim religion, and emerging regions had significantly negative CIs, indicating these variables were more prevalent among the poor.

The contribution of each determinant to the overall ECI of rotavirus vaccination is also shown in Table 2. The determinants included in the model explained 74% of the overall socio-economic inequality in rotavirus vaccine uptake. Antenatal care use (18.4%), exposure to media (12.8%), maternal educational level (9.7%), institutional delivery (8.1%), religion (6.1%), and urban residence (5.3%) were the major contributing factors to the overall inequality. Variables such as maternal age at birth, partner’s educational level, and child sex had relatively minimal contribution towards the inequality. About 26% of the overall inequality was due to the residuals (i.e., the inequality left unexplained).

## 4. Discussion

The result of the study shows that there was significant socioeconomic inequality in rotavirus vaccine uptake in Ethiopia to the disadvantage of children in the poorest households. Our finding is consistent with the results of several other previous studies [11,12,14,37,38]. A number of countries in Africa have documented a significant reduction in hospitalization associated with rotavirus and all-cause gastroenteritis following the introduction of rotavirus vaccine into their immunization program [8]. However, as children from the poorest household are at greatest risk of rotavirus infection, assuring equality in rotavirus vaccine uptake by increasing the vaccine uptake among this group of children can enhance the observed overall health gain. A model based study in the 25 Gavi, the Vaccine Alliance, supported countries showed that if the rotavirus vaccine uptake is equal between the richest and poorest quintiles, the number of rotavirus-associated deaths among the poorest quantile could increase by 89%. The study also estimated that the total number of lives saved increased by 38% as a result of equity-oriented rotavirus vaccine uptake [39]. It is eminent that identifying factors contributing to the inequalities is a preliminary step to design appropriate policy measures that can potentially reduce the observed socioeconomic inequalities.

The decomposition analysis shows that most of the inequalities in the uptake of rotavirus vaccine were explained by inequalities in maternal health services utilization (i.e., antenatal care, institutional delivery, and postnatal care). Overall, they explained about 30% of the total inequality in rotavirus vaccination uptake. Given that immunization services are commonly obtained from the same health facility where maternal health care is provided, maternal health care utilization can be considered as a proxy indicator for the accessibility of health facilities where immunization services are provided. Thus, the observed inequality could be associated with the disparity in access to the immunization service. On the other hand, although maternal healthcare services are supposed to be provided free of charge at public health facilities in Ethiopia, mothers often make payments at the point of service use [40,41,42,43]. This can hinder the utilization of the services among the majority of the economically disadvantaged groups who are mainly dependent on public health facilities for their health care needs. Previous studies in Ethiopia also identified indirect costs, poor quality of the service, and lack of supplies and equipment at public health facilities as barriers to get the service [40,41,44,45,46,47]. Moreover, since less affluent mothers cannot afford much more expensive services from private providers, unlike their wealthier counterparts, this further widens the utilization gap across the socioeconomic strata. Previous studies in Ethiopia have documented the positive role of maternal health care utilization on childhood immunization [48,49]. Our result also confirms the finding of other studies where antenatal care use was identified as a major contributing factor for the inequality in childhood immunization [12,50]. From a policy perspective, targeted intervention is required to enhance maternal healthcare utilization in Ethiopia among the economically disadvantaged group to mitigate inequality in the vaccination uptake. Financial incentives in the form of conditional cash transfer and voucher scheme for poor households might be potential interventions to improve maternal health care demand in developing countries.

Media can influence people’s health-seeking behavior by informing, motivating, and reminding them of certain healthcare services [51,52]. The positive association between immunization coverage and media use was documented in various studies [53,54,55]. In our study, exposure to media explained about 13% of the measured rotavirus vaccine uptake inequality. Expanding community radio programs could be an important platforms to address the inequality associated with access to media in Ethiopia.

The maternal educational level appears to explain a considerable portion of inequality in rotavirus vaccination (8.89%); as confirmed in earlier studies [14,56]. In addition to improving the level of health literacy [57,58], better education for mothers could contribute to financial freedom, self-confidence, and increased decision-making power for their own as well as their children’s healthcare needs. In the last decades, remarkable progress has been made in promoting education in Ethiopia. Primary education is provided free of charge and the enrolment rate increased from 20% to 86% between 1994 and 2015 [59,60]. There has been also a remarkable growth in terms of access to both secondary and tertiary education in Ethiopia [61]. However, about half of (48%) women aged between 15–19 years old have no formal education. Almost three in four women in the poorest households have no formal education, compared to one in five women in the wealthiest households [17]. Therefore, in addition to closing the gap in educational attainment over the long term, it is necessary to set up an immediate system to inform mothers about the importance of childhood immunization programs. A number of previous studies in different parts of Ethiopia have reported maternal educational level as a significant determinant for immunization coverage [62,63,64,65].

It is also noted that some of the socioeconomic inequalities in the uptake of rotavirus vaccine were attributed to religion (6.1%). Based on the finding in this study, a prominent contribution mainly springs from Muslim religion, as the variable was significantly negatively associated with the uptake of the vaccine and disproportionally concentrated among the mothers from the less wealthy households. It can, therefore, be proposed that the participation of Muslim religious leaders in the promotion of vaccination could reduce inequalities in Ethiopia. The positive role of religious leaders in rising immunization coverage among children has been highlighted in previous studies [66]. Indeed, given that the vast majority of the Ethiopian population is religious and respectful of religious leaders, the overall potential of religious institutions to improve the national immunization coverage needs to be closely explored in the future.

Our study also showed that urban residence was a major contributor to inequality. This could be partly explained by better access to immunization services in urban areas compared to rural areas that are more likely to be characterized by hard-to-reach communities. The urban-rural disparity in childhood immunization uptake has also been reported in other studies [63,67].

Furthermore, inequality in distance to a health facility, which can be a proxy measure of physical access to vaccination site, is one of the contributing factors to the inequality of rotavirus vaccination in Ethiopia. Traveling longer distances could increase transport costs and productivity losses, thereby raising the economic burden on mothers in the poorest household and impacting the uptake of vaccines among their children. Previous studies from Ethiopia and other developing countries identified long distance from a health facility and a time taken to reach there as a significant deterrent for immunization uptake [68,69,70,71]. Expanding alternative vaccination delivery modalities, such as outreach and mobile strategies, could improve the physical access and mitigate vaccination inequalities associated with long travel distance.

The findings of this study also highlighted that about 27% of the socioeconomic related inequality of rotavirus vaccination was left unexplained. This is not surprising because our model took into account mostly demand-side determinants. However, various studies documented several supply-side factors as a predictor for immunization coverage [9,10,72,73,74], such as cold chain management, vaccine availability, and adequacy of staffing which we are not able to include in our model due to lack of such information in the dataset we used. Moreover, there may be other demand-side factors that could be associated with underlining socio-economic inequality and that need to be examined in future studies. A relevant share of unexplained inequality was also reported in a previous study with a similar design as ours [75].

Beyond measuring the socioeconomic inequality, this study facilitates the understanding of the underlying factors, enabling policymakers to take these as a guide to formulate a relevant strategy that addresses the inequality. However, this study has some limitations. First, an asset-based wealth index was used as a proxy measure of socioeconomic status in this study as the EDHS lack direct measures such as income, expenditure, or consumption. However, it was shown that the asset-based approach is a suitable alternative method for inequality studies in the absence of direct measures of socioeconomic status [76]. Second, as data on supply-side determinants is lacking in EDHS, their effect on the inequality was not investigated in our study. In addition, only rotavirus vaccine was considered in the current study. Hence, supply-side determinants and other vaccines which are already included in the national immunization program should be taken into account during further study to get a comprehensive picture.

## 5. Conclusions

Our research has shown significant pro-wealthy inequities in rotavirus vaccination in Ethiopia. Policy makers need to be aware of the existing disparity and adopt equity-oriented policies to benefit socioeconomically marginalized groups. We suggest that targeted interventions towards increasing maternal health care service and educational attainments among economically disadvantaged mothers can significantly reduce inequality in rotavirus vaccine uptake in Ethiopia.

## Figures and Tables

**Table 1:** Characteristics of children aged 12–23 months by sex, residence place, region, and wealth index in Ethiopia (source: EDHS 2016; N ^‡^ = 2004).

Variable	N ^‡^ (%)
Sex of child	
Male	926 (46.2)
Female	1078 (53.8)
Place of Residence	
Rural	1772 (88.4)
Urban	232 (11.6)
Region	
Established regions	1821 (90.9)
Emerging regions	122 (6.0)
Addis Ababa and Dire Dawa	62 (3.1)
Wealth quintile *	
Poorest	504 (25.2)
Poorer	396 (19.8)
Middle	450 (22.4)
Richer	366 (18.3)
Richest	288 (14.4)

‡ Sampling weights were applied; * the wealth index is grouped into quintile.

**Table 2:** Decomposition analysis of the Erreygers’ normalized concentration index for rotavirus vaccine uptake in Ethiopia.

Determinant	Coefficient	CI	Contribution	% Contribution
Mother’s age in years (reference is 20–34)				0.1
15–19	−0.021 (0.0904)	−0.018 (0.0029) ***	0.000 (0.0000) ***	0.1 (0.0131) ***
35–49	−0.001 (0.0438)	0.009 (0.0011) ***	0.000 (0.0000) *	0.0 (0.0179) *
Maternal educational level (reference is no education)				9.7
Primary	0.084 (0.0428)	0.104 (0.0009) ***	0.010 (0.0002) ***	3.8 (0.0716) ***
Secondary and higher	0.069 (0.0766)	0.744 (0.0011) ***	0.017 (0.0006) ***	6.2 (0.2271) ***
Mother’s with employment status of working	−0.006 (0.0359)	0.075 (0.0007) ***	−0.001 (0.0002) ***	−0.2 (0.0630) ***
Religion (reference is Orthodox)				6.1
Protestant	−0.065 (0.0448)	0.102 (0.0012) ***	−0.006 (0.0002) ***	−2.3 (0.0646) ***
Muslim	−0.112 (0.0421) **	−0.140 (0.0008) ***	0.025 (0.0003) ***	9.3 (0.1362) ***
Others	0.035 (0.1077)	−0.446 (0.0034) ***	−0.002 (0.0002) ***	−0.8 (0.0990) ***
Administrative regions (reference is Established regions)				3.8
Emerging regions	−0.027 (0.0413)	−0.439 (0.0011) ***	0.003 (0.0001) ***	1.0 (0.0521) ***
Addis Ababa and Dire Dawa	0.074 (0.0535)	0.836 (0.0006) ***	0.007 (0.0002) ***	2.8 (0.0696) ***
Female child	0.004 (0.0346)	0.020 (0.0006) ***	0.000 (0.0001) ***	0.1 (0.0243) ***
Antenatal care use	0.179 (0.0409) ***	0.108 (0.0005) ***	0.049 (0.0004) ***	18.4 (0.1700) ***
Institutional delivery	0.056 (0.0433)	0.265 (0.0008) ***	0.021 (0.0005) ***	8.1 (0.2098) ***
Urban residence	0.040 (0.0692)	0.735 (0.0015) ***	0.014 (0.0007) ***	5.3 (0.2807) ***
Exposure to media	0.090 (0.0528)	0.510 (0.0012) ***	0.034 (0.0007) ***	12.8 (0.2540) ***
Postnatal care use	0.099 (0.0551)	0.308 (0.0015) ***	0.010 (0.0002) ***	3.7 (0.0708) ***
A mother not considered distance to a health facility as a big problem	0.039 (0.0360)	0.212 (0.0007) ***	0.013 (0.0004) ***	4.6 (0.1486) ***
Partner’s educational level (reference is no education)				1.3
Primary	0.020 (0.0436)	0.057 (0.0008) ***	0.002 (0.0001) ***	0.8 (0.0511) ***
Secondary and higher	0.004 (0.0662)	0.583 (0.0012) ***	0.002 (0.0006) **	0.5 (0.2420) *
Residual	0.410 (0.0533) ***			25.9
ECI decomposed	0.270 (0.0012) ***			

CI: concentration index; ECI: Erreygers’ normalized concentration index; Bootstrapped SEs are presented in parenthesis; *, **, *** *p*-value < 0.05, <0.01, and <0.001, respectively.
